# Individual Pharmacotherapy Management (IPM-II) for Patient and Drug Safety in Polypharmacy via Clinical Electronic Health Record Is Associated with Significant Fall Prevention

**DOI:** 10.3390/ph17121587

**Published:** 2024-11-25

**Authors:** Ursula Wolf, Luise Drewas, Hassan Ghadir, Christian Bauer, Lars Becherer, Karl-Stefan Delank, Rüdiger Neef

**Affiliations:** 1 Pharmacotherapy Management, University Hospital Halle (Saale), Martin Luther University Halle-Wittenberg, 06120 Halle (Saale), Germany; 2 Internal Medicine Clinic II, Martha-Maria Hospital Halle-Dölau, 06120 Halle (Saale), Germany; 3 Department of Internal Medicine/Cardiology, Johanniter-Hospital Geesthacht, 21502 Geesthacht, Germany; 4 Department of Orthopaedics, Trauma and Reconstructive Surgery, University Hospital Halle (Saale), Martin Luther University Halle-Wittenberg, 06120 Halle (Saale), Germany; 5 Department of Orthopaedics, Trauma and Reconstructive Surgery, Division of Geriatric Traumatology, University Hospital Halle (Saale), Martin Luther University Halle-Wittenberg, 06120 Halle (Saale), Germany

**Keywords:** fall prevention, aged, in-hospital falls, polypharmacy, inpatient Electronic Health Record (EHR), pathophysiological conditions, Individual Pharmacotherapy Management (IPM), patient safety, drug safety

## Abstract

Background/Objectives: Falls and fractures are emerging as a near-pandemic and major global health concern, placing an enormous burden on ageing patients and public health economies. Despite the high risk of polypharmacy in the elderly patients, falls are usually attributed to age-related changes. For the “Individual Pharmacotherapy Management (IPM)” established at the University Hospital Halle, the IPM medication adjustments and their association with in-hospital fall prevention were analysed. Methods: On the basis of the most updated digital overall patient view via his inpatient electronic health record (EHR), IPM adapts each drug’s Summary of Product Characteristics to the patient’s condition. A retrospective pre-post intervention study in geriatric traumatology on ≥70 years old patients compared 200 patients before IPM implementation (CG) with 204 patients from the IPM intervention period (IG) for the entire medication list, organ, cardiovascular and vital functions and fall risk parameters. Results: Statistically similar baseline data allowed a comparison of the average 80-year-old patient with a mean of 11.1 ± 4.9 (CG) versus 10.4 ± 3.6 (IG) medications. The IPM adjusted for drug-drug interactions, drug-disease interactions, overdoses, anticholinergic burden, adverse drug reactions, esp. from opioids inducing increased intrasynaptic serotonin, psychotropic drugs, benzodiazepines, contraindications and missing prescriptions. IPM was associated with a significant reduction in in-hospital falls from 18 (9%) in CG to 3 (1.5%) in IG, a number needed to treat of 14, relative risk reduction 83%, OR 0.17 [95% CI 0.04; 0.76], *p =* 0.021 in multivariable regression analysis. Factors associated with falls were antipsychotics, digitoxin, corticosteroids, Würzburg pain drip (combination of tramadol, metamizole, metoclopramide), head injury, cognitive impairment and aspects of the Huhn Fall Risk Scale including urinary catheter. Conclusion: The results indicate medication risks constitute a major iatrogenic cause of falls in this population and support the use of EHR-based IPM in standard care for the prevention of falls in the elderly and for patient and drug safety. In terms of global efforts, IPM contributes to the running WHO and United Nations Decade of Healthy Ageing (2021–2030).

## 1. Introduction

Despite decades of worldwide recognition and global efforts, falls and fractures are increasingly emerging as an almost pandemic public health concern, placing an enormous burden on ageing patients and public health economies. They represent one of the most important global health issues, as evidenced by the literature on the subject [1,2,3,4,5,6]. In 1990 already, the total number of hip fractures in men and women was estimated at 338,000 and 917,000, respectively, for a total of 1.26 million. Assuming no change in the age- and gender-specific incidence, the number of hip fractures was calculated to approximately double to 2.6 million by 2025 and to 4.5 million by 2050. Based on modest assumptions about secular trends, the incidence of hip fractures in 2050 could range from 7.3 to 21.3 million [1]. Reflecting these earlier projections, the Global Burden Disease Fracture Collaborators even published in 2021 that more than 10 million hip fractures occur in people aged 55+ worldwide, based on 2019 data, and the top three regions by age-standardised prevalence overall rate of fractures were Australasia, central Europe, and Eastern Europe [2]. For future prognosis, the recent 2023 study with a forecast on the global epidemiology of hip fracture and trends in incidence rates estimated that the total annual number of hip fractures will almost double between 2018 and 2050 [3]. The World Health Organization (WHO) has published a series of increasingly concerning reports and fact sheets on this problem [4,5,6].

The pursuit of risk assessments and prevention strategies to reduce the incidence of falls and their consequences in older age, primarily fractures and cerebral haemorrhages, has been ongoing for almost three decades. These strategies have been developed using multifactorial [7,8,9,10] and interdisciplinary approaches and are increasingly promoted internationally through broad-based consensus guidelines [11,12,13,14]. The focus is on strength, balance and exercise training, administration of calcium, vitamin D though its effect being questioned [15,16,17], bisphosphonates and RANK ligand monoclonal antibodies, nutritional aspects, staff and family education, and even fall apps, hip protectors [18,19], fall mats, brightness and spatial sensors, 3-axis acceleration and 3D position sensors, which are all part of this prospering market.

The issue of the more cost-effective elimination of drug-related iatrogenic causes of falls due to avoidable polypharmacy risks is still not receiving the attention it deserves. The cumulative fall risks determined over 12 years in over 65,800 medication reviews conducted by the author led to this intervention analysis. In addition to the personal suffering of patients, particularly in cases of loss of independent living and the need for admission to a nursing home, the consequences of falls represent an increasing demographic, personnel and financial challenge for the healthcare system: The global population is ageing rapidly. The number of people aged 65 and over is set to double from 1 billion in 2019 to 2.1 billion by 2050, while the number of people aged 80 and over is predicted to triple worldwide [20]. This coincides with a growing shortage of caregivers and imposes almost unmanageable economic costs on healthcare systems. Approximately one-third of individuals aged 65 and above experience a severe fall at least once a year, with one in two individuals aged 80 and above experiencing a severe fall on an annual basis [21]. The direct financial burden of a femoral neck fracture is estimated to be approximately €20,000, exclusive of indirect costs associated with care [22]. Projections indicate that the annual direct costs of hip fractures in Germany are anticipated to rise from €2.77 billion to €3.85 billion by 2030 [23].

Alerted, the United Nations has proclaimed the period between 2021 and 2030 the ‘Decade for Healthy Ageing’ and has commissioned the WHO to lead its implementation [24]. To enhance the quality of life for the elderly, it is imperative to prioritise the implementation of comprehensive primary preventive measures. The Harvard Medical Practice Study [25], which established as early as 1991 that iatrogenic drug-related harm is correlated with increasing age and that quality standards and sanctions for non-compliance are called for, is still not being consistently addressed despite the increase in risky polypharmacy. The results of studies on the efficacy of medication adjustment to reduce falls are not satisfactory. However, there have been at least some promising positive studies in selected patient cohorts and selected drug groups, e.g., [26,27], also from the Nurse-Led Medicines’ Monitoring for Patients with Dementia in Care Homes [28]. The majority of studies focus on a limited range of medications [29,30,31,32,33], such as lists of beneficial or detrimental drugs for the elderly, without delving into the specific clinical circumstances of the individual patient and his or her organ functions, respecting vulnerability and drug degradation capacity and requirements in the acute situation. 

There is an increasing availability of electronic health records (EHR) of the patients to be treated, especially for the inpatients. An EHR provides the opportunity to take into account the patient’s accurate and up-to-date condition in order to adjust each of his or her medications individually. To the best of our knowledge, no other published study has employed the EHR as the sole basis for an accurate, patient-adapted medication review for the prevention of polypharmacy-related risks, such as cognitive decline, organ deterioration, haemorrhages or falls.

Against this broad background of deficiencies, Individual Pharmacotherapy Management (IPM) was designed and implemented at the University Hospital Halle (UKH), resulting in a significant reduction of complications such as delirium and renal impairment [34,35,36,37,38]. The objective of the present study was twofold: firstly, to describe the pathophysiological conditions and associated medication risks identified by the IPM and subsequent adaptations; secondly, to analyse the associated impact of the IPM on fall prevention and further risk factors associated with in-hospital falls.

## 2. Results

### 2.1. Group Comparison for Baselines

**Figure 1:**
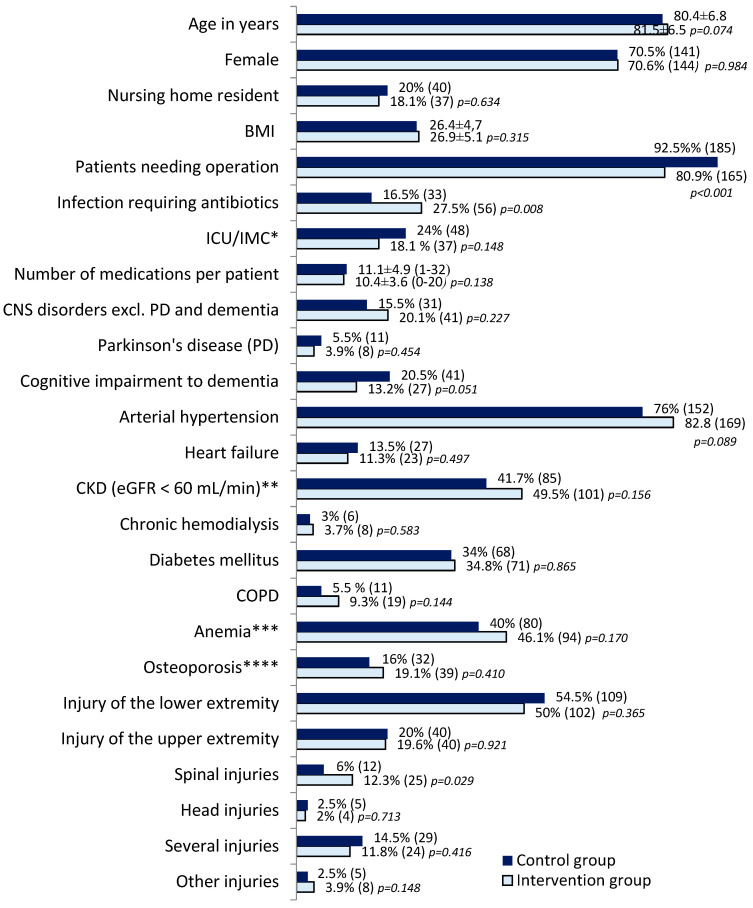
Group comparison of CG (200 patients) and intervention group (204 patients) with respect to baseline characteristics, inpatient variables, selected diagnoses and admission injury (percentages for direct comparison (absolute numbers), except mean ± SD for age, BMI and number of medications) and *p*-values from nonparametric testing. * Transient stay in the intensive care unit (ICU) or intermediate care (IMC). ** Chronic kidney disease (CKD). *** Anaemia: haemoglobin in women < 7.1 mmol/L, in men < 8.4 mmol/L. **** Osteoporosis as far as assessed and ICD-coded.

The high degree of matching between the two groups in the baseline data in terms of age, gender, type of residence, BMI, and the majority of the concomitant diagnoses considered, as well as the incidence of hospitalisation injuries, was regarded as an essential prerequisite for comparability (Figure 1). The elderly patients were predominantly female, and 80% were community-dwelling. The leading cause of admission was a fracture of the lower extremity in both groups. Admissions following falls in patients with severe head injury and intracerebral haemorrhage are not included here as they are referred to other specialist disciplines.

Over 80% of patients underwent surgical intervention. The extent rate of surgical need was higher in the control group (CG), and in the intervention group (IG), there were more spinal injuries. There were also more infectious diseases requiring antibiotics in the IG. The prevalence of pre-existing central nervous system (CNS) disorders, such as stroke, was higher in the IG, while Parkinson’s disease and cognitive impairment were more common in the CG.

### 2.2. IPM-Identified Pathophysiological Conditions with Iatrogenic Drug Risks and Interventions

*Anaemia* (46% IG, 40% CG) (Figure 1) indicated further diagnostics and causal therapy, for example, in renal anaemia as a secondary disease condition. In cases of macrocytic anaemia with an increased mean corpuscular volume (MCV), as from metformin- or proton pump inhibitor (PPI)-associated vitamin B12 deficiency, targeted substitution or, in cases of confirmed shortage, folic acid supplementation was provided. In microcytic hypochromic form, partly associated with direct oral anticoagulants (DOACs), further diagnostic assessments and treatment of iron deficiency anaemia for suspected occult bleeding were undertaken. DOACs frequently demonstrated unconsidered pharmacodynamic drug-drug interactions (DDIs), particularly an elevated risk of haemorrhage upon concomitant administration of selective serotonin reuptake inhibitors/selective serotonin-norepinephrine reuptake inhibitors (SSRIs/SSNRIs) or low-dose acetylsalicylic acid (ASA). Furthermore, DOAC overdoses in renal insufficiency, less frequent pharmacokinetic DDIs and contraindications with potent cytochrome P450 system 3A4 (CYP3A4) or p-glycoprotein (p-GP) inhibitors or liver dysfunction required adjustments.

Based on updated eGFR values, 50% of IG patients (42% CG) exhibited impaired renal function, classified as *chronic kidney disease (CKD)* stage 3 or above according to the KDIGO (Kidney Disease Improving Global Outcomes) criteria. The BIS formula from the Berlin Initiative Study [39,40] demonstrated that the estimated glomerular filtration rate (eGFR) MDRD may overestimate renal function in stage CKD3a, underscoring the necessity of considering the applied GFR estimate in interpretation. The prevalence of renal insufficiency and CKD stage >2 was underdiagnosed in >50% of cases, frequently not considered in medication dosing, including antibiotics, and coded in only <50% of physician letters [35]. The standard operating procedure (SOP) that was implemented was to withdraw non-steroidal anti-inflammatory drugs (NSAIDs) in favour of time-limited metamizole. However, there were exceptions in case of some rare prophylactic NSAID indications for heterotopic ossification, which were excluded if there was severe renal insufficiency and/or if there were concomitant drug risks with ACE inhibitors or sartans, and particularly always avoiding so-called “triple whammy” with add-on diuretics, furthermore [41].

The frequent *overdosing of proton pump inhibitor (PPI)* prophylaxis, for example, the administration of pantoprazole 40 mg rather than the recommended 20 mg dose per day, prompted a hospital-wide best practice PPI reduction project.

Manifest *hyponatraemia* (10.3% IG, 6.5% CG) constitutes a further example of probably drug-induced syndrome of inappropriate antidiuretic hormone secretion (SIADH). In such cases, withdrawal of the offending agent, for example, hydrochlorothiazide, was often indicated, particularly in high-risk, long-term outpatient combinations with loop diuretics for sequential nephron blockade. Cumulative risks of hyponatraemia were frequently identified in patients receiving combination therapy with ACE inhibitors or sartans with aldosterone antagonists, diuretics, PPIs and SSRI/SSNRI or mirtazapine. In such cases, medication adjustments were required on an individual basis in accordance with the patient’s specific medical needs.

*Atrioventricular block (AV block)* I–III° was identified in 21% of electronically available electrocardiograms (ECGs) in both groups without further enumeration of the single severity frequencies. For *AV blocks or bradycardic arrhythmias* from combination risks of beta-blockers (also consider timolol eye drops) with predominantly non-eGFR-adapted moxonidine or under metoprolol or nebivolol with CYP2D6-inhibiting melperone, citalopram, sertraline, as well as acetylcholinesterase-inhibiting antidementia drugs, such as donepezil or galantamine, or with the ADP/P2Y inhibitor ticagrelor, and in the case of iatrogenic drug-induced risks, for example, those associated with antipsychotics, or thyreogenic risks of *tachycardic arrhythmias*, medication changes were targeted. The polypharmacy regimen of elderly patients often involved a risk of *QTc prolongation* from different drugs in a cumulative manner, which needed to be focused on and excluded. This was often the case with the antipsychotic and antidepressant classes. Maintaining serum magnesium and potassium levels in the upper range, in addition to QTc follow-up, is a preventive measure when there is an unavoidable transient drug of concern.

The up to 7–9-fold reduced renal elimination rate of *memantine* through *alkaline urine* [42], which is actually more common in elderly patients due to reduced protein intake or high doses of PPIs or urinary tract infections with Proteus mirabilis, demanded more consequent consideration.

Individual adjustment of antihypertensive single agents was required in the presence of clinical symptoms of orthostasis and dizziness for patients with predominantly *hypotensive blood pressure* values (RR systolic < 120 mmHg) (18.5% CG, 12.8% IG).

The administration of carvedilol was found to be inadequate for a relevant number of patients affected by *chronic obstructive pulmonary disease (COPD),* necessitating a switch to cardioselective beta-blockers.

*Pregabalin* was often prescribed on an outpatient basis for generalised anxiety disorder and neuropathic pain in accordance with the guidelines for diagnostics and therapy in neurology [43]. However, the potential for adverse effects was not fully considered despite the outlined risks and SmPC warnings of dizziness, falls and cognitive dysfunction, especially in the elderly on polypharmacy and on further concomitant central nervous system-influencing drugs such as opioids or benzodiazepines [44]. Furthermore, pregabalin was frequently dispensed without an appropriate eGFR dose adjustment, necessitating reassessment at the latest after the serious outpatient fall with hospitalisation for fracture.

In addition, required age-adjusted simvastatin dose or reduced simvastatin due to amlodipine interactions were identified to need the corrections. Preference should be given to *statins* with higher efficacy and fewer interactions.

Contraindications to *bisphosphonates* in hypocalcaemia or renal insufficiency were under-recognised. *Vitamin D* prophylaxis in patients on long-term corticosteroid or aromatase inhibitor therapy or with renal osteoporosis was also often unmatched.

*Protein-rich supplementation* in cases of malnutrition, low total protein, and low serum albumin benefited drugs with high protein binding. High-calorie therapy in severe dystrophy required careful monitoring of blood glucose levels.

**Figure 2:**
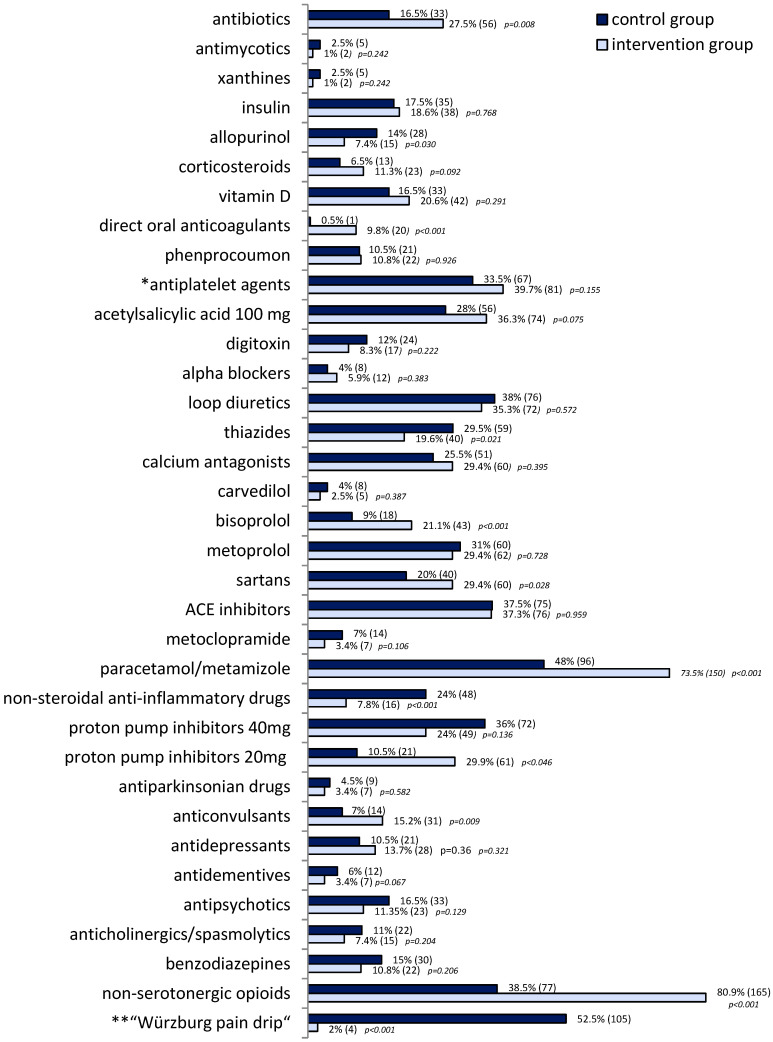
Prescription rates (percentage for direct comparison and (absolute numbers)) of drugs/drug groups in control (200 patients) and intervention group (204 patients). Initiated stepwise deprescribing of antipsychotics, antidepressants, benzodiazepines and alpha-blockers is not shown here due to incomplete discontinuation of the required stepwise process during the short-term surgical stay. * ADP/P2Y inhibitors or low dose ASA ** Würzburg pain drip = intravenous or peroral application of serotonergic tramadol, metamizole and metoclopramide.

The *adjusted total number of inpatient medications*, including postoperative analgesics, thromboprophylaxis and eventually antimicrobials, was 11.1 ± 4.9 in the CG and 10.4 ± 3.6 in the IG. Due to the short length of hospital stay and the medication doses not shown, the dose tapering process (*deprescribing*) of psychotropic drugs, benzodiazepines, alpha-blockers and cumulative anticholinergic burden (ACB) is not comprehensively depicted in the IG. The distribution pattern of administered drugs/drug groups (Figure 2) largely reflects the list of leading drugs in the Drug Prescription Report [45]. However, the spectrum of outpatient medication lists of these very elderly patients with *polypharmacy* due to multimorbidity was often extended by concomitant psychotropic drugs, opioids and sedatives. Their cumulative risk of dizziness and falls from drug-iatrogenic pathophysiological disorders was often considered to be the cause of the hospitalisation-relevant and severe fall events.

In addition to the wide range of the intraindividual IPM-related medication adjustments described above, the IPM strategy always included six evidence-based fall and cognitive risk aspects with the deprescription/reduction of

1. psychotropics, alpha-blockers and benzodiazepines [46];
2. combinations with iatrogenic-additive long-QTc risk;
3. anticholinergic components [47] partly directly from e.g., various urological spasmolytics, antivertiginosa and indirectly from cumulative anticholinergic adverse drug reactions (ADRs);
4. additive serotonergic risks, which can be confused with delirium, e.g., often due to SSRIs and SSNRIs, especially in contraindicated combination with serotonergic opioids; therefore, switch from tramadol and fentanyl to non-serotonergic hydromorphone [48,49,50];
5. always exclusion of overdoses and pharmacokinetic DDIs, and
6. severe or cumulative ADRs through pharmacodynamic DDIs.

### 2.3. Associations

**Figure 3:**
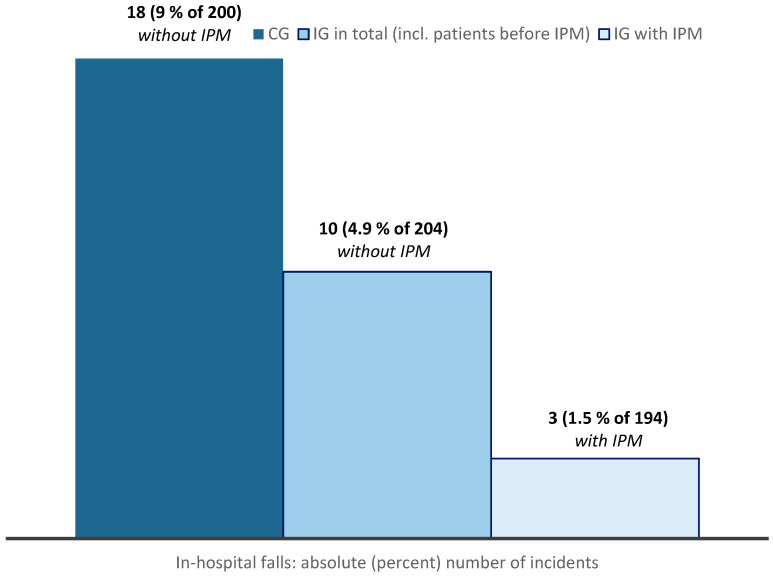
Reduction in in-hospital falls in geriatric traumatology during the intervention period. Comparison of the absolute incidence (percentage) of in-hospital falls in the CG, the IG group as a whole (including patients without IPM who fell before the 14-day IPM visit) and the IG with IPM. The 10 patients without IPM who fell before the fortnightly visit during the intervention period underline the individual intervention effect of IPM and indicate a considerably lower potential overarching group effect of the intervention due to adjusted Standard Operating Procedures (SOPs) only, e.g., in analgesic therapy.

IPM was associated with a reduction in in-hospital falls from 18 (9%) to 3 (1.5%), OR = 0.16 [95% CI 0.05; 0.55], *p =* 0.004 (Figure 3). The number needed to treat (NNT) was 14 (13.3), with an absolute risk reduction of 7.5%, and the relative risk reduction was 83%. Associated with in-hospital falls were antipsychotics, digitoxin, corticosteroids, the “Würzburg pain drip” (co-administration of serotonergic tramadol, metamizole, metoclopramide), admission for head injury, cognitive impairment, and aspects of the Fall Risk Scale by Huhn [51] including urinary catheter/enterostoma (Table 1). The reduction in falls associated with IPM remained significant in multivariable regression analysis with a precision estimate of OR = 0.17 [95% CI 0.04; 0.76], *p =* 0.021 (Table 1).

## 3. Discussion

### 3.1. IPM-Associated Impact and IPM-Specified Risks in the Literature Context

The risk reduction associated with IPM indicates the possibility of effective fall prevention in the most vulnerable elderly patients. Always depending on the baseline risk in the control group [52], the number needed to treat (NNT) achieved is remarkable, especially as a result of a completely prophylactic and extremely cost-effective intervention compared to the enormous socio-economic financial burden of treating drug-related falls and fractures. Numerous pathophysiological conditions have been identified in these elderly patients, which require the elimination of the corresponding iatrogenic drug risks.

In addition to the statistical gender distribution in the older age group [53], the prevalence in women reflects the increased risk of fracture after menopause. It is estimated that one in two women aged >50 years will suffer an osteoporosis-related fracture, compared with one in five men [54], and the risk increases with age. Osteoporosis and sarcopenia promote frailty and the age-related risk of falls and fractures, and vice versa [55,56,57,58,59]. In this context, inappropriate prescribing of PPIs has been identified, which is known to increase the risk of osteoporosis, falls and fractures, especially in the long term [60,61,62]. Analogous to another of the authors’ studies [63], an appropriate indication for the widely used therapeutic doses of PPIs was most often missed in the outpatient elderly, and the prophylactic regimen, e.g., 20 mg/day for pantoprazole, was almost typically overdosed by 40 mg/day, similar to clinical evidence. As a consequence, hospital-wide PPI educational sessions were conducted at the UKH as part of an intervention project to interrupt the overprescribing of PPIs.

The geriatric trauma patients exhibited typical polypharmacy due to multimorbidity, which now is required to be considered as an integrated, holistic approach as a guideline [64]. Medication safety in polypharmacy has also become an issue for WHO to focus on globally [65]. Medication management of this elderly patient group requires a comprehensive evaluation of the medication list adapted to the individual patient’s condition according to the IPM concept.

There are numerous intervention projects [66,67,68,69,70] with more general adjustment strategies, including the deprescribing of fall-risk-increasing drugs (FRIDs) [67], some of which were less or even not successful. In addition to assessing the deprescribing of avoidable substances, the overall individual focus, as evidenced by the IPM conducted, must always be on the entire risk spectrum, including overdoses, e.g., as a result of underdiagnosed chronic kidney disease being a worldwide problem [35,71], individual and, in particular, cumulative ADRs and DDIs, and drug contraindications despite prescribing in accordance with drug approvals or guidelines. There have been extensive analyses of fall risk factors [72,73,74,75,76] and different approaches to fall prevention, with [77] or without effects [78], implementing strategies besides the obviously most important pharmacological aspect. Thus, the results of the different interventions did not reach the preventive effect associated with the comprehensive IPM adjustment of the individual patient’s medication list, in which each individual medication is adapted to the updated and defined clinical scores of the patient by means of an internal medicine synopsis. This procedure respects the most detailed real-time patient condition while contributing to the entire circuit that affects the intraindividual bioavailability of a single drug. As such, IPM also considers drug-disease interactions and represents an individualised intervention aimed at achieving the best risk-benefit ratio for each drug prescription.

CYP2D6 inhibition, with a documented increase in fall injuries [79], contributed to the most common pharmacokinetic DDIs in polypharmacy in elderly patients, e.g., citalopram, sertraline and/or melperone as frequent inhibitors of the CYP2D6 metabolism of concomitant ß-blockers such as metoprolol and nebivolol. Apart from gradually reducing the psychotropic drugs involved, e.g., SSRIs and SSNRIs with questionable efficacy in mild and moderate depression [80], switching to bisoprolol, which is pharmacokinetically inert in this respect, has also been a way out, as indicated by its significantly increased rate in the IG.

Antivertiginosa, which are suspiciously promoted at geriatric congresses, must be scrutinised as a prescribing cascade and avoided because of its often anticholinergic, cognition-impairing and fall-increasing ADRs.

The Huhn Fall Risk Scale [51], which primarily considers intrinsic fall risks, including cognitive dysfunction, proved to be prognostically valuable and showed overlap with the Hospital Frailty Risk Score [81]. For urinary catheters as another risk factor, there has been some further evidence by Mion and colleagues investigating the risk of injurious falls in hospitalised patients [75].

The identified fall-associated drugs and drug groups entirely confirmed their SmPC-reported ADRs [82] in this context and decades and updates of literature data, e.g., for digitoxin already prescribed in 1999 by Leipzig et al. [83] and in 2018 by de Vries et al. [84], for antipsychotics, e.g., by Landi et al. in 2005 [85] and Seppala et al. in 2018 [86]. The Würzburg pain drip, a combined administration of tramadol, metamizole, metoclopramide, intravenous or peroral, revealed a significant association with falls in the univariable regression analysis and remained clinically relevant within the multivariable analysis. It was almost completely withdrawn through the IPM in the IG. Tramadol was substituted by non-serotonergic hydromorphone through an SOP in this elderly patient group. From the tramadol SmPC [87], as well as from literature data, there are various ADRs to be notified of, including the risk of fracture documented in a female patient population ≥75 years of age [88]. And any CYP2D6-inhibitor may reduce the tramadol analgesic effect that might eventually lead to consecutive dose elevation because of impaired activation of tramadol as a prodrug. Metoclopramide should be avoided in elderly patients for its ADRs, including drowsiness, dizziness, somnolence, hypotension, parkinsonian effects and acute dystonic reactions. Additionally, the required metoclopramide dose adaptation to renal function was frequently not considered, thus increasing the exposure and the ADRs. In Germany, metamizole, requiring monitoring for its potentially myelosuppressive effect, has remained the alternative agent for postoperative analgesia [89] in vulnerable elderly patients, who are already often prescribed ACE inhibitors or sartans and diuretics or even suffering from CKD, to prevent further renal injury or acute renal failure, particularly in high-risk conditions of the “triple whammy” with add-on NSAIDs [41]. Effective analgesic synergy was achieved with metamizole plus the non-serotonergic opioid hydromorphone, established as a SOP as well.

### 3.2. Way Out Measures Needed for Overarching Challenges

The 2019 WHO report on medication safety in polypharmacy states: “A comprehensive medication review is a multidisciplinary activity whereby the risks and benefits of each medicine are considered … It optimises the use of medicines for each individual patient…Polypharmacy can put the patient at risk of adverse drug events and drug interactions when not used appropriately.” ([65], p. 7). The intentionally comprehensive individual IPM concept fulfils the requirements of the global challenges that the WHO continues to address, and it has been established at UKH for more than a decade. Its acceptance is very positive, and the interdisciplinary networking does not require time-consuming activities for the attending physicians, as each IPM result is presented in rapid telemedical digital or phone communication or via interdisciplinary patient visits.

The benefits of an EHR presented here are an example of the analogue potential of the EHR in this cross-sectoral neglected context of ambulatory prevention. IPM can be performed digitally from outside on the basis of the EHR, without the need for on-site personnel competences or valences. The reproducibility and interoperability of IPM were also verified by pharmacists. Since the method is applicable online with an EHR it suggests a potentially similar EHR value in the context of high-risk polypharmacy in the outpatient setting to accurately adjust medications to the patient’s overall condition, taking into account the impaired organ and body functions of the elderly [90] for the most accurate consecutive adjustment. The entire IPM process takes an average of only 6.5 min per patient with a daily routine and a complete EHR. It enables seamless, digital, real-time interdisciplinary networking and is applicable in all online environments. The resulting recommendations for attending colleagues are communicated simultaneously via telemedicine or, in the case of geriatric trauma patients, via interdisciplinary rounds that visit each patient. Pharmacists who were instructed in IPM were readily able to reproduce the results of the IPM checks according to the defined patient and drug scores. Advanced medical students also did not require long training. The time spent by the attending physician, who is informed of the necessary medication adjustments, is about 2 min per patient and does not require any special training.

The European Union Geriatric Medicine Society (EUGMS) recommends that fracture prevention should include both measures to prevent falls and optimisation of bone health [91]. It should be noted that the latter can also be affected by medication, e.g., psychotropic medication-induced mobility impairment, corticosteroid-associated osteoporosis, or PPIs, which are mostly inappropriately indicated, overdosed [63], and administered for far too long [92]. Investigating the pathophysiological mechanisms, Desai and colleagues recently demonstrated a negative effect of pantoprazole use on Ca^2+^ and Mg^2+^ levels, which could affect the melastatin-like transient receptor potential 7 (TRPM7) channel involved in bone cell proliferation and thus alter the TRPM7-mediated bone remodelling process [61]. Geriatricians, in particular, should demand a good standard and take responsibility for individual medication analysis in addition to considering positive and negative lists [14]. Identification of specific medication risks, e.g., as investigated in oropharyngeal dysphagia [93,94], should be part of every geriatric assessment since psychotropic drugs, opioids or benzodiazepines, among others, individually and especially when used concomitantly, have an iatrogenic negative effect on both cognitive reactivity and muscle strength of a patient, thus already signalling the need to adjust his medication [95].

Impaired organ function in old age must be taken into account not only in prescribing but also in drug development and approval [90].

The inpatient EHR proved to be an efficient basis for an up-to-date and complete overview of IPM patient scores with a minimal time expenditure. In addition, interdisciplinary IPM networking is also likely to have a pharmacological educational effect on the treating physicians, promoting their risk/benefit awareness when prescribing medications across different medical specialities.

The results, which are to be substantiated by a randomised controlled trial (RCT), recommend a cross-sectoral integration of this structured IPM into an overall therapeutic concept, which should be demanded and supported by the health insurance funds, also as a premise of the much propagated “holistic patient care”.

### 3.3. Limitations

Although the patient groups are structurally nearly identical, this is a retrospective study with its inherent disadvantages compared to a prospective RCT. The wide range of variables, with the potential for missing data in both groups due to incomplete documentation, requires a large number of cases. In the absence of data from an IPM pilot project, it was not possible to calculate an adequate sample size. In relation and with reference to the limited cut of the total number of cases collected, it was drugs and drug groups rather than also dosages that were recorded, with the exception of PPIs. The differentiation of the subsumed agents requires further studies in extended populations. However, the sample size was already rather extense, and for the CG and IG comparison, the required and presented *p*-values may be less meaningful than η_p_^2^ values from nonparametric testing in terms of documenting clinically relevant differences. While clinically relevant differences may be statistically insignificant, they may be worth considering. This issue is increasingly being addressed in medical studies, and so reference to mere statistical significance should not be used as the sole cut-off or basis for further attention.

The study population consisted mainly of older patients who had already suffered a serious fall outside the hospital. As a history of falls has been demonstrated to be an independent risk factor for falling, this must be considered a bias predisposing our patient samples to re-fall and thus increased risk of in-hospital falls [96]. The overall number of history falls was not counted.

The wide time span over which the patients in the two groups were compared might have been affected by variations in the setting. However, to the best of our knowledge, we were able to exclude modifications in medical, nursing and physiotherapy care. There were no noticeable changes in the type and number of medications from the patients’ pre-existing ambulatory medication lists, with the exception of DOACs.

There is currently no additional routine analysis at the UKH for further intraindividual genotype impact in terms of metabolic polymorphism, such as in the metabolising enzymes CYP3A4, CYP2D6 and the multidrug efflux pump P-glycoprotein, encoded by MDR-1. The IPM does not routinely include foods and substances related to complementary and alternative medicine (CAM), such as dietary supplements, herbs and other manufactured ingredients, although this has been shown to be of further relevance, especially in outpatients, e.g., in a cancer study sample, with a likelihood of interaction of up to 37% in the case of CAM supplements and 29% of all patients for foods [97,98].

In-hospital falls were not stratified by time of onset, and intraoperative blood loss was not considered. The consequences of the in-hospital falls and their impact on length of stay were not part of this analysis.

## 4. Materials and Methods

### 4.1. Setting, Study Design and Data Collection

A fortnightly interdisciplinary ward round has been implemented for patients aged ≥70 years in the geriatric traumatology department of the University Hospital Halle (UKH) since 2011. This is conducted by the IPM physician and the geriatric traumatologists, accompanied by the nursing staff, geriatricians, physiotherapists and ergotherapists, and social services specialists. The basis for this is a medication analysis. The continuity of collaboration between the head of trauma surgery in geriatric traumatology and the IPM physician specialised in internal medicine with expertise in clinical pharmacology, as well as the intervention strategies with respect to the patients’ homogeneous injury and fracture spectra, ensures long-term procedural consistency. No changes were made that were relevant to the setting, including those to trauma surgery procedures, applied care standards, physiotherapy, or anaesthesia.

In a clinically controlled pre-post study, 404 inpatients were retrospectively compared. The control group (CG) comprised 200 patients and was sampled from 2/2009 to 12/2010, closest to the start of the IPM intervention. The IPM intervention group (IG) comprised 204 patients and was sampled from 5/2012 to 8/2016 from the at random preserved printed part of the IPM medication reviews with detailed notifications for adjustments. The recruitment process was conducted in a blinded manner with regard to the outcome of “in-hospital fall”. Due to the two-week interval between IPM visits, there were also single patients in the IG period who had fallen prior to the IPM intervention. In-hospital falls were recorded in a uniform manner by nursing staff over the entire observation period on the digital documentation form set up for this purpose.

The anonymised data collection (Table 2) from the ORBIS digital hospital information system included a wide range of laboratory parameters, with the estimated glomerular filtration rate (eGFR) at follow-up (using the Modification of Diet Renal Disease (MDRD) formula at the time of the study), chronic and acute diagnoses relevant to the questions under investigation, electronically recorded electrocardiograms (ECGs), a fall risk scale according to Huhn [51], current injury, surgery, transient postoperative intensive care (ITS) and intermediate care (IMC) stay, as well as the patient’s comprehensive medication list, including outpatient and inpatient adjusted medications.

### 4.2. IPM Intervention

**Figure 4:**
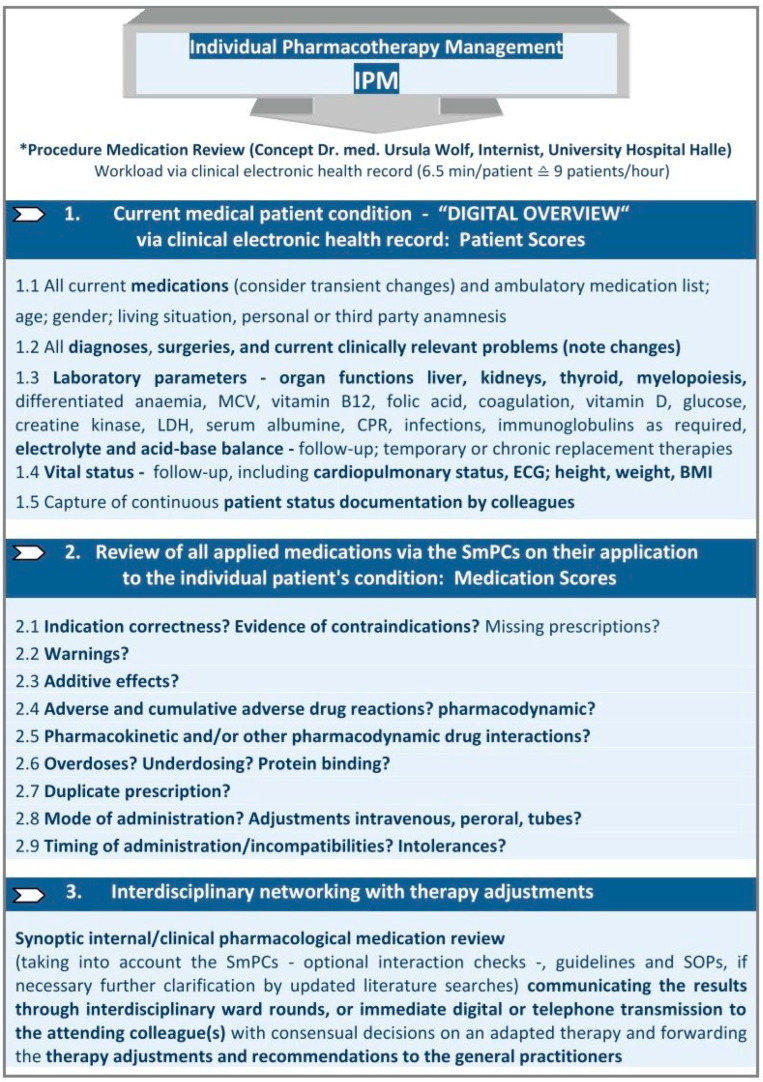
Comprehensive, reproducible Individual Pharmacotherapy Management to identify and counteract individual pathophysiological patient conditions which may be iatrogenically induced by drugs. * IPM (applied patient and medication scores) based on the inpatient Electronic Health Record (EHR) and Summaries of Product Characteristics (SmPCs), conceptualised, implemented and practised by Ursula Wolf, MD, Head of Pharmacotherapy Management Department, Specialist in Internal Medicine with expertise in Clinical Pharmacology, performed >65,800 individual medication reviews for polypharmacy in elderly trauma, dementia, transplant and intensive care patients. The IPM physician applies the SmPCs by matching their evidence-based defined risks to the digitally captured individual patient scores providing the necessary “comprehensive patient view”.

At the UKH, the IPM procedure (Figure 4) has been conceptualised and established for more than a decade and has proven to be significantly associated with the successful prevention of the risks of polypharmacy [34,35,36,37,38]. The IPM has been implemented by a single physician in the disciplines where polypharmacy could be most critical for the patients and their pre-existing vulnerable condition: the geriatric traumatology department, the intensive care units and the organ and stem cell transplantation departments of the UKH.

It is conducted as a synthesis of internal medicine and clinical pharmacology in accordance with the clinical training of the responsible IPM physician. It provides continuous interdisciplinary networking based on the clinical electronic health record (EHR) for each patient. The reproducible IPM protocol refers to the most accurate current clinical status of the patient in terms of organ function and vital parameters, as determined by the most recent assessments. In order to ensure that the prescribed medications are aligned with the patient’s overall diagnoses and acute clinical situation, and to account for factors such as drug degradation and excretion capacities, as well as elevated drug exposure and the potential ADRs from real-time manifest pharmacokinetic DDIs, a comprehensive analysis of the entire medication list is essential. The list is analysed on the basis of the SmPCs [82] for ADRs, including pharmacodynamic DDIs, with particular attention paid to their cumulative presence in polypharmacy, contraindications, warnings and dosage regimen. Furthermore, the analysis encompasses missing prescriptions. Additional tools are employed, for instance, in different types of renal replacement therapy, either intermittent or continuous [99,100,101], and for further verification of the interaction [102,103] in cases where there are still remaining questions, extending to the performance of literature searches. Accordingly, the entire “medication scores” are evaluated in accordance with the established national and international guidelines while taking into account the defined digital “patient scores” from the updated inpatient EHR. During the ward rounds for geriatric trauma patients, the individual medication changes are implemented through an interdisciplinary consensus following the presentation of identified risks. These changes are then transmitted as a recommendation to the attending physician in the discharge/transfer letter.

In other medical disciplines, such as intensive care and organ and stem cell transplantation, the IPM networking has been implemented through rapid daily telemedical phone or digital communication. The complete digital view of the patient via the EHR enables the IPM procedure to be carried out with some routine in just 6.5 min per patient, whether the IPM is performed daily or once. As with intensive care or transplant patients receiving the same IPM, an individual’s condition may change abruptly and require completely new, close-meshed, even daily IPM due to a change in medication or disease complications and organ drug degradation capacities involvement. IPM is performed autonomously, although for the present study in the same hospital. The patient’s hospital doctors are informed of the necessary adjustments to be made according to the IPM results. Thus, the IPM can be applied the same way, provided an EHR, centrally online for external patients and hospitals as approved. In particular, the clearly defined IPM measure can also be administered and completed by pharmacists on an interprofessional basis, as validated by both clinical and community pharmacists.

### 4.3. Statistics

Statistical analysis in cooperation with the Institute of Medical Epidemiology, Biometry and Informatics based on Microsoft Excel 2016 for anonymous data collection from the hospital information system EHRs and SPSS Statistics 24. The parameters within the different categories collected corresponded to variables relevant to the research objectives based on clinical-empirical and literature data. Descriptive analysis was used to compare the two groups (CG 200 patients and IG 204 patients), including nonparametric testing by one factorial analysis of variance and chi-square test, respectively, providing *p*-values. Dummy variables were generated to assess clinically relevant nominally categorised variables. Logistic regression analysis including the entire study population to assess patients with fall events in CG (200) plus IG (196 = 204 minus 10 patients from the intervention period who had a fall event prior to IPM due to the 14-day interval between IPM visits) was applied to identify independent factors associated with in-hospital falls. Univariable logistic regression analysis was performed on all 115 variables to analyse their association with the dependent variable in-hospital fall. The multivariable regression analysis model was generated from the resulting variables of the univariable logistic regression analysis with a *p*-value ≤ 0.05 to adjust for robust covariables and using a 95% confidence interval.

## 5. Conclusions and Outlook

According to the association results, the IPM could make a decisive and overdue contribution to patient and drug therapy safety, with a still largely untapped prevention potential. It comprehensively covers the broad spectrum of pathophysiological conditions with drug therapy risks due to polypharmacy in the elderly at a most individual level by means of the applied patient and medication scores. The intentionally comprehensive IPM concept fulfils the requirements of the global challenges that the WHO continues to address. As already approved by pharmacists, it is applicable centrally online from anywhere provided an EHR for the patient scores. The high and even extensive concurrent effectiveness compared to other intervention projects, as the same IPM drug adjustments were simultaneously associated with a 90% reduction in delirium [34] and a 100% reduction in the progression of renal impairment [35], is most likely due to the patient-centred concept with the strongly individualised approach. Mandatory IPM integration for geriatric patients in primary care in Germany through guidelines by the Federal Joint Committee (G-BA) would be urgently recommended to relieve the burden of falls and fractures on the individual patient, which can threaten the independence of older adults and cause a cascade of individual problems and socio-economic health care system challenges. 

The analysed pathophysiological disorders with fall risks and overlapping cognitive risks from the many years of real-world experience of currently >65,800 IPMs in polypharmacy are to be integrated into an electronic and compatible patient documentation programme that is accessible across sectors.

The continued passive acceptance of the mandatory categorisation of ‘death after a fall’ as a ‘non-natural’ cause of death in Germany reinforces the need for an active paradigm shift. An ICD classification with the appropriate term “Suspected Drug-Associated Fall” could help to raise awareness and responsibility for this seriously neglected medical problem in order to improve the quality and efficiency of healthcare for our growing patient population. Fall prevention concerns physicians at all levels and specialities, and furthermore pharmacists and nurses. To start fall prevention the important step earlier before hospital admission, it is imperative that all healthcare professionals involved in the medication process proactively identify and address any potential drug-related adverse changes in patients at the earliest stage. This was the consequent next engagement of the IPM author, who subsequently conducted interprofessional educational training workshops on the identified IPM polypharmacy risks in the cross-sectoral outpatient setting, the fall and fracture predetermining ambulatory sector [104].

## Figures and Tables

**Table 1:** Variables associated with the outcome “in-hospital fall”. 1. Variables significantly associated with fall events (univariable regression analysis, *n* = 394 patients). 2. Relevant and significant associations with fall events in the multivariable regression analysis, including the significant variables from the univariable analysis. To further analyse their associations with the dependent variable, the outcome of in-hospital falls, the entire study group of 394 patients (excluding 10 patients from the IG period who fell before receiving an IPM intervention) was included and univariable logistic regression analysis was performed on all 115 variables to analyse their association with an in-hospital fall. The multivariable regression analysis model was generated from the resulting variables of the univariable logistic regression analysis with a *p*-value ≤ 0.05 to adjust for robust covariables and using a 95% confidence interval (CI).

1. Univariable regression analysis	*p*-Value	OR	95-%-CI	Number (*n* = 394)
Individual Pharmacotherapy Management (IPM)	0.004	0.16	0.05–0.55	194
Cognitive dysfunction to dementia	0.008	3.48	1.38–8.79	64
Admission with head injury	0.041	5.50	1.07–28.31	9
Antipsychotics	0.012	3.39	1.30–8.81	55
Digitoxin	0.007	3.99	1.45–10.95	40
Corticosteroids	0.017	3.71	1.27–10.84	34
“Würzburg pain drip”	0.041	2.52	1.04–6.11	109
Fall risk scale according to Huhn				384
Temporarily confused/disorientated	0.005	4.38	1.55–12.43	40
Gait unsteady/unstable	0.026	6.38	1.25–32.51	69
Walking impaired	0.029	5.54	1.19–25.78	131
Bladder catheter/enterostoma	0.011	4.69	1.43–15.42	90
**2. Multivariable regression analysis**	***p*** **-Value**	**OR**	**95-%-CI**	**Number (*n* = 394)**
Individual Pharmacotherapy Management (IPM)	0.021	0.17	0.04–0.76	194
Cognitive dysfunction to dementia	0.297	1.98	0.55–7.09	64
Admission with head injury	0.009	15.82	2.02–123.81	9
Antipsychotics	0.774	1.21	0.33–4.46	55
Digitoxin	0.157	2.41	0.71–8.14	40
Corticosteroids	0.002	8.28	2.12–32.36	34
“Würzburg pain drip”	0.503	1.45	0.49–4.30	109
Fall risk scale according to Huhn				384
Temporarily confused/disorientated	0.104	2.80	0.81–9.72	40
Gait unsteady/unstable	0.265	2.24	0.54–9.22	69
Walking impaired	0.026	4.06	1.18–13.99	131
Bladder catheter/enterostoma	0.009	4.01	1.41–11.36	90

**Table 2:** Categories and variables (*n* = 115) collected from the inpatient electronic health record for association measures with in-hospital falls and other outcomes of further IPM intervention analyses.

**Demographics:** age, gender, type of residence (home/nursing home)
**Vital parameters at admission:** BMI, blood pressure (day course), heart rate (day course)
**Continuous and acute medication:** number of drugs, angiotensin converting enzyme inhibitors (ACE inhibitors), sartans, calcium antagonists, differentiated ß-blockers, α-blockers, antibiotics, antifungals, antiarrhythmics, antidementives, anticonvulsants, different oral anticoagulants, bisphosphonates, different antiplatelet drugs, different diuretics, antipsychotics, antidepressants, St. John’s wort, oral antidiabetics, insulin, antiparkinsonian drugs, benzodiazepines, proton pump inhibitors (PPI) (incl. dosage), ophthalmics, anticholinergics, spasmolytics, muscle relaxants, opioids, “Würzburger pain drip” ^1^, tramadol, nonsteroidal anti-inflammatory drugs (NSAIDs), further analgesic agents, antiemetics, thyroid hormones, xanthines, uricosurics, uricostats, statins, vitamin D, corticosteroids, other drugs (e.g., hormones, cytostatics)
**Laboratory parameters at admission:** blood count, electrolytes, inflammation parameters, renal function parameters (eGFR) during course of stay, myoglobin, coagulation parameters, urinalysis
**ECG (if available online):** rhythm, frequency, QTc interval ^2^, atrioventricular block (AV block)
**Diagnoses ^3^:** arterial hypertension, heart failure, complicating delirium, cognitive impairment to dementia, Parkinson’s disease, further central nervous system (CNS) disorders, chronic obstructive pulmonary disease (COPD), diabetes mellitus, osteoporosis, chronic kidney disease (CKD) according to updated actual eGFR MDRD values
**Additional course aspects:** changes in laboratory findings, blood pressure, heart rate, temperature, cognitive changes/disturbances, pain symptoms and profile, other subjective complaints of the patient
**Other parameters:** acute admission injury, operation, transient stay in IMC ^4^ or ICU ^5^, hemodialysis, length of hospital stay, perioperative infections, fall risk scale according to Huhn (0–31 points, broken down according to: age, mental status, excretion, history of falls, gait/balance, activities, medication, alcohol), pacemaker, defibrillator, infections requiring antibiotics, contrast medium application

^1^ Combination of tramadol, metamizole, and metoclopramide administered intravenously or partially orally. ^2^ time from the start of the *Q wave* to the end of the *T wave* (measurement on ECG). ^3^ coded in the hospital discharge letter except for chronic kidney disease. ^4^ Intermediate care. ^5^ Intensive care unit.

## Data Availability

The dataset generated and analysed for the current study is available from the corresponding author upon reasonable request.
